# Effects of ethyl acetate extract of *Salsola collina* on brain-gut peptides and interstitial cells of gastric Cajal in rats with diabetic gastroparesis 

**DOI:** 10.22038/ijbms.2020.43521.10223

**Published:** 2020-09

**Authors:** Xinling Zhao, Hongbo Wang, Ziwei Zhang, Hong Jin, Yanling Gong

**Affiliations:** 1 Department of Pharmacy, College of Chemical Engineering, Qingdao University of Science and Technology, Qingdao, Shandong, China; 2 Department of Gastroenterology Surgery, Jimo People’s Hospital, Qingdao, Shandong, China

**Keywords:** Brain-gut peptides, Cajal interstitial cells, Diabetic gastroparesis, Ethyl acetate extract, Gastric motility, Salsola collina

## Abstract

**Objective(s):**

Effects of ethyl acetate extract of *Salsola collina* (EES) on brain-gut peptides and interstitial cells of gastric Cajal in rats with diabetic gastroparesis were explored.

**Materials and Methods:**

Rats were divided into six groups: normal control group (NC), diabetic gastroparesis model group (DGP), low, medium, and high dose of EES groups (LES, MES, and HES, respectively), and metoclopramide positive group (MPG). DGP rats were induced by streptozotocin (STZ) combined with a high-sugar-high-fat diet. The gastric emptying was measured by the phenol red labeling method. Enzyme-linked immunosorbent assay (ELISA) was employed to determine the concentrations of serum ghrelin, gastrin (GAS), somatostatin (SS), and vasoactive intestinal peptide (VIP). The expressions of c-Kit and its natural ligand stem cell factor (SCF) in gastric tissues were determined by Western blot and immunofluorescence.

**Results:**

Gastric emptying rate increased in a different degree after intervention by EES, among which MES and HES groups showed a significant effect (compared with DGP, *P*<0.01) and the HES group was equivalent to the MPG group; serum ghrelin and content of serum GAS increased while SS and VIP decreased (compared with the DGP group, *P*<0.05 or *P*<0.01); c-Kit and SCF protein expressions in gastric tissue increased (compared with DGP group, *P*<0.05 or *P*<0.01).

**Conclusion:**

EES significantly improved gastric emptying by regulating gastrointestinal hormone excretion and c-Kit/SCF signaling pathway. Our study provides a pharmacological basis for the use of the EES in the treatment of DGP. However, the detailed molecular mechanism remains to be clarified.

## Introduction

Diabetes mellitus (DM) is a major metabolic chronic disease with a global prevalence rate of nearly 400 million people (1). Long-term DM often leads to systemic complications, including cardiovascular disease, neuropathy, retinopathy, kidney disease, and gastrointestinal disease. Diabetic gastroparesis (DGP), characterized by satiety, nausea, vomiting, postprandial fullness, abdominal pain, and delayed gastric emptying without signs of mechanical obstruction (2), is a common complication occurring in approximately 30-50% of patients with type 1 and type 2 DM (3). Although DGP is not malignant, it inhibits the absorption of nutrients and oral anti-diabetic drugs, which may interfere with glycemic control and lead to inefficient treatment of DM (4). The pathogenesis of DGP is still under investigation which is believed to be multifactorial including hyperglycemia, intestinal nervous system (ENS) damage, brain-gut peptide disorders, myopathy, interstitial cells of Cajal (ICC) loss, etc. (5-7). All above factors are involved in gastric movement and emptying by regulating gastrointestinal pacing and smooth muscle contraction. ICC is a non-neuronal cell in the muscularis propria, which controls gastrointestinal motility primarily by controlling electrical pacemaker activity (8). The protein c-Kit expressed by ICC is a receptor of tyrosine kinase expressed by ICCs oncogenes. Normal expression of the c-Kit gene is essential for maintaining a normal signaling pathway that regulates gastric contraction (9), and the presence of c-Kit immunoreactivity is useful for identifying ICCs and recognizing their distribution in the digestive tract. In addition, the survival and function of ICCs is dependent on stem cell factor (SCF, a c-Kit ligand), which activates c-Kit and induces multiple downstream signaling pathways (10). Studies have shown that SCF-Kit signaling is important for maintaining ICC phenotype, proliferation, and differentiation (11).

*S. collina *is a genus of *Salsoloideae*, widely distributed in Central and Southwestern Asia, the Mediterranean, and North Africa. It is an important desert vegetation and grows in saline-alkali areas (12). Studies on its chemical composition have shown that *S. collina* mainly contains alkaloids, flavonoids, sterols, and organic acids (13, 14). Pharmacological experiments have also shown that it has significant antihypertensive effects (15) and central inhibition (16), and alkaloids are one of the material basis of its antihypertensive effect. As a chronic disease, DGP must be treated with long-term medication. The existing research shows that current medications such as metoclopramide, domperidone, and erythromycin have side effects in the treatment of DGP, which may result in new damages (17, 18). Nowadays, the extraction of active ingredients from plants for the treatment of chronic diseases has become a new trend. Our previous studies have shown that the extract of *S. collina* can promote gastrointestinal motility and has hypoglycemic activity *in vitro* (19, 20). Therefore, we speculate whether *S. collina* has a certain effect on DGP. Based on the screening of the active ingredients, ethyl acetate extract of *Salsola collina *(EES) was prepared and its effect on gastric emptying in DGP was observed. Due to the involvement of brain-gut peptides and ICC in the pathogenesis of DGP, the effect of EES on the secretion of brain-gut peptides and ICC regulation were explored. The possible mechanism might lay the foundation for the development of the relevant drugs for the extract of *S. collina*.

## Materials and Methods

***Materials and reagents***

Female Sprague-Dawley rats were provided by Qingdao Daren Fortune Animal Technology Co., Ltd. and weighed 150 g-180 g (animal license number: SCXK (Beijing) 2016-0002). The temperature of animal pegs was controlled at 22±2 ^°^C, and the humidity at 55±10% with 12 hr light-dark cycle. All rats were fed adaptively for 7 days. The experiment was approved by the Animal Protection and Use Ethics Committee of Qingdao University of Science and Technology.

The basic feed was purchased from Qingdao Daren Fortune Animal Technology Co., Ltd.; high sugar and high-fat feed references literature formula (21): 66.5% basic feed, 20% sucrose, 10% lard, 1.5% cholesterol, 1% sodium cholate, and 1% egg yolk powder.

The EES was prepared by the ethanol reflux method. The process conditions were as follows: the ratio of material to liquid was 20 ml.g^-1^, the concentration of ethanol was 70%, and the extraction time was 3 times and the reflux time was 1.5 hr, 1.5 hr, and 1 hr, respectively. The extract was collected and concentrated into concrete; it was dispersed in distilled water and repeatedly extracted with an equal volume of ethyl acetate. The upper layer solution was collected, concentrated, and dried using a vacuum dryer (DZF-6020, Shandong Boko Bio-Industry, China) to obtain the EES.

***HPLC***

High performance liquid Chromatographic analysis was performed using an Agilent 1200 Series LC system (Agilent Technologies, Inc., Japan) consisting of a quaternary pump, a manual injector, a column oven, and a degasser. HPLC was carried out using a C18 guard column (Phenomenex C18, Japan) prior to the analytical column of an Agilent C18 column (4.6 mm ×250 mm, 5 μm particle size, Japan). A mobile phase consisting of A: B-0.01% (v/v) phosphoric acid: methanol was used at a flow rate of 1 ml/min. Gradient elution according to the following elution procedure (22, 23): 0-9 min, 95% A; 10-15 min, 90% A; 16-20 min, 85% A; 21-30 min, 80% A; 31-40 min, 70% A; 41-60 min, 60% A. The temperature of the injector was room temperature, the injection volume was 5 μl, and the detection wavelength was 290 nm.

***Animal grouping and establishment of DGP rat model***

Eighty rats were used in this experiment. Sixteen rats were randomly divided into normal control (NC) group, and the remaining rats were used to establish a rat model of DGP. The model rats were intraperitoneally injected with freshly prepared streptozotocin (50 mg.kg^-1^), and the NC group was injected with the same volume of physiological saline. After 72 hr, rat tail vein blood was collected, and fasting blood glucose (FBG) was measured using a blood glucose meter. Diabetes was defined when FBG≥16.7 mmol.l^-1^ (lasting for 2 weeks) (24). Subsequently, diabetic rats were fed irregularly with high-sugar-high-fat (HSHF) diets on odd-day mornings and even-day afternoons, respectively (25). Rats in the NC group were given a normal diet. After 10 weeks, 8 rats were randomly taken from the NC group and the diabetic group to measure gastric emptying. The differences in gastric emptying and blood glucose were observed to confirm the establishment of diabetic gastroparesis.

Forty rats with successful establishment of DGP were randomly divided into 5 groups: diabetic gastroparesis model control group (DGP), low dose (16 mg/(kg.d)), medium dose (32 mg/(kg.d)) and high dose (64 mg/(kg.d)) ethyl acetate extract of *S. collina* groups (LES, MES, and HES, respectively) and metoclopramide (Yun Peng Pharmaceutical, China) positive control group (MPG, 170 mg/(k.d)). The intragastric administration was continued for 4 weeks.

***Blood sample and tissue collection***

Following final administration, the rats were fasted overnight, weighed, and administered with 1.5 ml of a 0.05% phenol red solution. One hour later, the rats were anesthetized by intraperitoneal injection of sodium pentobarbital (65 mg/kg). After half an hour, a blood sample was taken by cardiac puncture and centrifuged at 3500 rpm/min for 15 min to prepare the serum and stored at -20 ^°^C. Then the rats were sacrificed by decapitation, and the cardia and pylorus were ligated. The whole stomach was taken out, cut along the greater curvature, and washed with 0.9% sodium chloride solution. Gastric contents were collected and the volume was adjusted to 20 ml. A portion of gastric tissue was embedded in paraffin and sectioned for c-Kit immunofluorescence staining. Another portion of the gastric tissue was stored frozen at -80 ^°^C for Western blot analysis.

***Gastric emptying***

The gastric contents were added to 20 ml of 0.5 mol/l NaOH and mixed well. After quiescence for 1 hr, 5 ml of the supernatant was taken out, added with 0.5 ml of trichloroacetic acid (20% w/v) for deproteinization, and centrifuged at 3500 r/min for 10 min. The absorbance value of the supernatant was measured with a spectrophotometer at 560 nm. 18 ml of distilled water, 20 ml of 0.5 mol/l NaOH, and 4 ml of trichloroacetic acid (20% w/v) were added to another 2 ml of the phenol red solution and mixed to measure the absorbance as a blank control (26).

Gastric emptying rate (%)= (1-(phenol red absorbance in gastric tissue homogenate)/(blank control phenol red absorbance))×100%

***Enzyme-linked immunosorbent assay (ELISA)***

The serum concentrations of ghrelin, gastrin (GAS), somatostatin (SS), and vasoactive intestinal peptide (VIP) were measured according to the ELISA kit instructions (NanJing JianCheng Bioengineering Inc., China) (27).

***Immunofluorescence staining***

The sections were deparaffinized in xylene, hydrated in a gradient ethanol solution, and the antigen was repaired using a citric acid repair solution. After being washed 3 times with PBS, goat serum was added and blocked in a wet box for 2 hr at room temperature. The blocking solution was removed, and rabbit anti-rat c-Kit (1:100, Absin Bioscience Inc., China) primary antibody was added to incubate overnight in a 4 ^°^C wet box and rinsed three times in PBS buffer. Fluorescent secondary antibody (cy3 label, 1:100, Beijing Bioss Biomart, China) was added and incubated in the dark box at room temperature for 2 hr. After anti-fluorescence quenching oil sheet, immunofluorescence staining was observed using a CKX53 fluorescence microscope (Olympus, Japan).

***Western blot analysis***

Rat gastric tissue was removed from a -80 ^°^C cryostat, and RIPA lysate (Beijing solarbio, China) was added. After lysis for 30 min, the homogenate was transferred to an Ep tube and centrifuged at 12000 rpm for 10 min at 4 ^°^C. Protein concentration was determined by the BCA protein quantification kit (Beijing solarbio, China). 30 μg of protein sample was transferred to a polyvinylidene fluoride membrane (PVDF membrane) on sodium dodecyl sulfate-polyacrylamide gel (SDS-PAGE) and blocked with 5% skim milk powder in TBST buffer for 2 hr at room temperature (25 ^°^C). Membranes were incubated with a specific rabbit anti-rat c-Kit antibody (1:1000) overnight at 4 ^°^C. All transferred membranes were washed 3 times in TBST buffer for 3 min and incubated with HRP-labeled goat anti-rabbit antibody (1:5000, Beijing Bioss Biomart, China) for 2 hr at room temperature. The substrate in the enhanced chemiluminescence kit was uniformly applied to the PVDF membrane and developed under a gel imaging system. The optical density of the protein bands was measured by autoradiography OptiQuant software and then normalized by Western blots parallel to GAPDH.

***Statistical analysis***

Data from the experiments are shown as quantitative data (±SD). Additionally, one-way ANOVA and Duncan’s new multiple range method were performed using SPSS 17.0 software. *P*<0.05 was considered to be statistically significant.

## Results

***HPLC analysis of EES***

**Figure 1:**
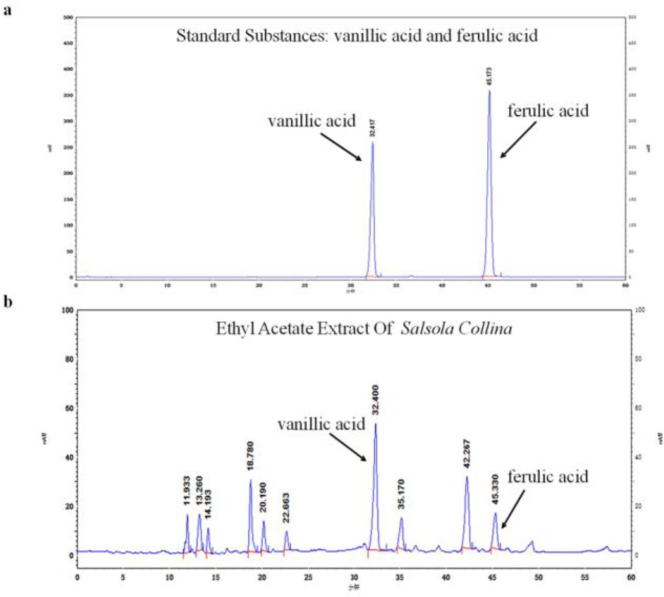
HPLC analysis of EES

The composition of the EES was shown in Figure 1, which has been identified as the main active ingredient to promote gastrointestinal motility. Vanillic acid and ferulic acid were detected in the EES by HPLC, which were speculated to be related to the activity to promote gastrointestinal motility. However, due to the complex composition of the ethyl acetate extract, it is necessary to continue the separation and identification of the active ingredients in order to provide a reference for the identification of gastrointestinal motility active substances.

***Effect of the EES on gastric emptying in DGP rats***

**Figure 2.**
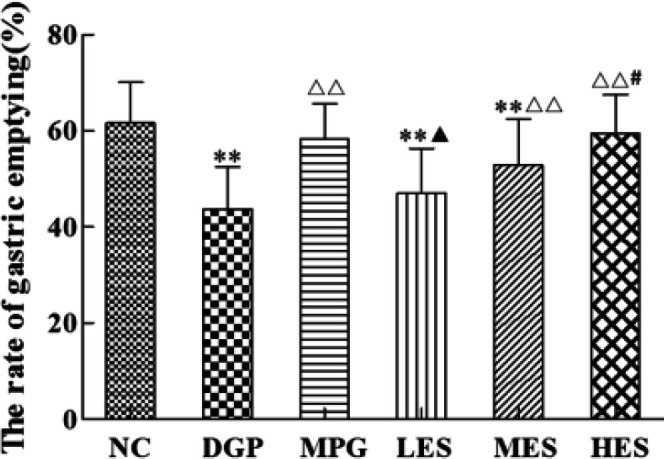
Effect of EES on gastric emptying in DGP rats

As shown in Figure 2, compared with the NC group, the gastric emptying rate in the DGP group significantly decreased (*P*<0.01), suggesting that the DGP model was established successfully. Compared with the DGP group, the gastric emptying rate in MES, HES and MPG groups increased significantly (*P*<0.01). Compared with the LES group, there was a significant improvement in gastric emptying in the HES group (*P*<0.05). The above results showed that the EES significantly promoted gastric emptying of DGP rats induced by STZ combined with the HSHF diet, exhibiting a certain alleviating effect.

***Effect of the EES on brain-gut peptides in DGP rats***

**Figure 3:**
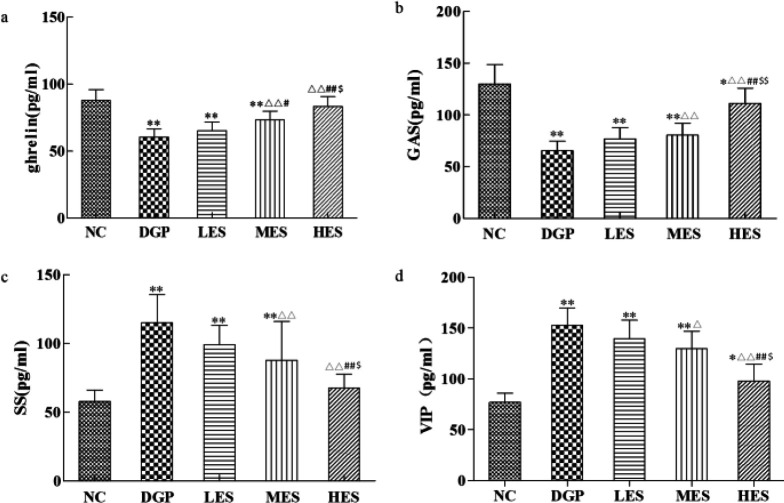
Effect of EES on brain-gut peptides in DGP rats

Our experimental results showed that serum ghrelin and GAS levels in the DGP group decreased while SS and VIP levels increased, showing a significant difference with the NC group (Figure 3, *P*<0.01), suggesting a disorder of brain-gut peptides in the DGP rats. Compared with the DGP rats, the levels of brain-gut peptides changed significantly after treatment with the EES (Figure 3, *P*<0.05 or *P*<0.01). Therefore, different doses of EES increased serum ghrelin and GAS levels, inhibited the secretion of SS and VIP, and thus improved gastrointestinal motility in the DGP rats.

***Effect of the EES on interstitial cells of cajal in the stomach of DGP rats***

**Figure 4:**
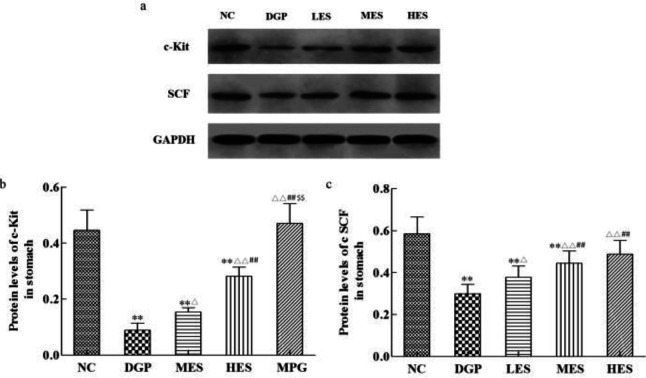
Effect of EES on protein expression of c-Kit and SCF in gastric tissue

**Figure 5:**
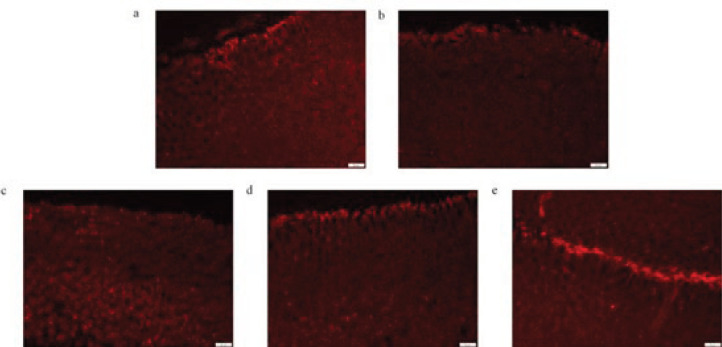
Effect of EES on immunoreactive positive reaction of Cajal interstitial cells in rat gastric tissue

In this study, rat gastric stroma Cajal cells were observed by Western blot analysis and immunofluorescence. Compared with the NC group, the c-Kit and SCF in the gastric tissue of the DGP rats significantly decreased (Figure 4, *P*<0.01). However, the expression of c-Kit and SCF protein in the LES, MES, and HES group significantly increased when compared with the DGP group (*P*<0.05 or *P*<0.01), showing a dose-dependent relationship. Furthermore, the fluorescence density in the DGP group was significantly lower than that in the NC group, while it increased to different degrees in the LES, MES, and HES group (Figure 5).

## Discussion

*S. collina* is an annual herb with strong adaptability and regenerability, drought tolerance and alkali resistance. The plant is rich in not only protein and trace elements, but also selenium, which is about twenty times that of ordinary food (28). The whole plant can be used as medicine. It is considered to have the effect of hepatoprotection, while also working as an antihypertensive as well as a laxative. Previous experiments have found that *S. collina *can promote small bowel peristalsis and accelerate emptying. In this experiment, a rat model of DGP was established by injecting STZ and feeding with an irregular HSHF diet for 10 weeks. The DGP rats were treated with EES, after which their gastric emptying, serum brain-gut peptide content, and gastric Cajal cells were measured. The results indicated that EES promoted gastric emptying in DGP rats, which may be related to its recovery of brain-gut peptide levels and regulation of c-kit/SCF signaling.

Streptozotocin (STZ) is reported to enter cells through glucose transporter 2 (GLUT2), which is toxic to pancreatic insulin-induced beta cells, leading to damage in tissues such as gastrointestinal and kidney (29). A small amount of STZ induces peripheral tissues to be insensitive to insulin, leading to destruction of islet β-cell function and induction of gastric dysmotility when combined to feed HSHF diet irregularly (30). The rats with DGP in our experiment showed symptoms such as nausea, weight loss, constipation, messy fur without luster, and apathy. After treatment with EES, vomiting reduced, bodyweight gradually recovered with stool molding and improved mental state.

In our experiments, serum brain-gut peptides were determined. Brain gut peptides is a group of high-activity substances secreted by the gastrointestinal mucosa and pancreatic endocrine cells, which can stimulate or inhibit gastrointestinal motility (31). Some examples of such substances are ghrelin, GAS, SS, and VIP. These peptides are released into the blood by endocrine cells and act on target organs to exert regulatory effects, as well as affect adjacent cells through paracrine. In addition, many brain-gut peptides act as neurotransmitters through nerve endings (32). Ghrelin and GAS stimulate the secretion of digestive juice and contraction of the gastrointestinal smooth muscle, promote the advancement of gastrointestinal contents, and trigger gastrointestinal motility. Conversely, SS inhibits the secretion of digestive juices such as pancreatic exocrine and bile secretion, weakens contraction of gastrointestinal smooth muscle, and delays gastrointestinal emptying. VIP inhibits gastrointestinal motility by impairing intestinal contraction and spontaneous pyloric contraction (33). Our results showed that EES could restore the level of serum brain-gut peptides to varying degrees, which is beneficial to alleviate the DGP.

There are many studies on DGP, but the mechanism is complicated. The effect of the c-Kit/SCF pathway on gastric Cajal cells was studied in our experiment. C-Kit is a transmembrane protein and its ligand stem cell factor (SCF) is produced by neuronal cells and smooth muscle cells. C-Kit and SCF promote the development and differentiation of ICC and maintains its normal physiological functions. The c-Kit tag indirectly reflects the number and density of ICC (34, 35). Therefore, a decrease in the number and function of ICC cells occurs in gastrointestinal motility disorders. When the c-Kit expression is down-regulated, the precursor cells develop into smooth muscle cells or fibroblast-like cells. However, the precursor cells differentiate into ICC when c-Kit continues to be expressed. It is reported that slow waves disappeared when c-Kit expression decreased (36). When the c-Kit expression is blocked by spontaneous c-Kit mutant animals, ICC almost disappears and loses slow-wave activity (37). Thus, low expression of c-Kit results in ICC loss and slow-wave attenuation, leading to gastric motility disorders. Since almost all ICCs express c-Kit, the distribution of ICCs can be examined by c-Kit immunohistochemical staining. We found that the total number of ICCs in the DGP rats decreased, and the destruction of the ICC network may attenuate the generation and spread of slow waves in the stomach and eventually lead to gastrointestinal dysmotility. The EES significantly increased the number of ICCs and restored the function of the ICC network by regulating the c-Kit/SCF signaling pathway, thereby promoting gastric motility in DGP. Combined with the above results together, we concluded that EES promoted gastric emptying in the DGP rats via regulating brain-gut peptide secretion and improving the ICC function. Although the pathogenesis of DGP is still under investigation, there are multiple factors and multiple mechanisms involved, among which brain-gut peptide disorders and ICC loss are two important factors. Traditionally, the advantage of traditional Chinese medicine is multi-target therapy and the EES is no exception. In our present study, it is revealed that EES targeted both the brain-gut peptides and ICC to alleviate DGP, showing a powerful and long-lasting effect with no significant side effect. However, the deficiency of traditional Chinese medicine is that the ingredients are complex and the active ingredients are not clear. Therefore, more mechanisms and effective substances need to be explored in our future study. 

a. HPLC chromatogram of vanillic acid and ferulic acid standards; b. HPLC chromatogram of the EES

EES: ethyl acetate extract of *Salsola collina*

Compared with the NC group, *P<0.05, **P<0.01; compared with the DGP group, *P*<0.05, *P*<0.01; compared with the MPG group, *P*<0.05,*P*<0.01; compared with the LES group, #*P*<0.05, ##*P*<0.01; compared with the MES group, $*P*<0.05, $$*P*<0.01

EES: ethyl acetate extract of *Salsola collina*; LES: low dose ethyl acetate extract of *S. collina; *MES: medium dose ethyl acetate extract of *S. collina;* HES: high dose ethyl acetate extract of *S. collina; *DGP: diabetic gastroparesis; NC: normal control; MPG: metoclopramide positive group (MPG)

a: the content of serum ghrelin; b: the content of serum gastrin (GAS); c: the content serum somatostatin (SS); d: the content of serum gastrin (GAS).

Compared with the NC group, *P<0.05, **P<0.01; compared with the DGP group, *P*<0.05, *P*<0.01; compared with the LES group, #*P*<0.05, ##*P*<0.01

EES: ethyl acetate extract of *Salsola collina*; LES: low dose ethyl acetate extract of *S. collina; *MES: medium dose ethyl acetate extract of *S. collina;* HES: high dose ethyl acetate extract of *S. collina; *DGP: diabetic gastroparesis; NC: normal control

a: Bands of c-Kit, SCF and GADPH; b: quantitative analysis of c-Kit in the stomach; c: quantitative analysis of SCF in the stomach

Compared with the NC group, *P<0.05, **P<0.01; compared with the model group, *P*<0.05, *P*<0.01; compared with the low dose group, #*P*<0.05, ##*P*<0.01

EES: ethyl acetate extract of *Salsola collina;* LES: low dose ethyl acetate extract of *S. collina; *MES: medium dose ethyl acetate extract of *S. collina;* HES: high dose ethyl acetate extract of *S. collina; *SCF: stem cell factor; NC: normal control; GADPH: glyceraldehyde-3-phosphate dehydrogenase

a: NC group; b: DGP group, c: LES group, d: MES group; e: HES group

EES: ethyl acetate extract of *Salsola collina*; DGP: diabetic gastroparesis; LES: low dose ethyl acetate extract of *S. collina; *MES: medium dose ethyl acetate extract of *S. collina;* HES: high dose ethyl acetate extract of *S. collina; *NC: normal control

## Conclusion

EES significantly promoted gastric emptying in the DGP rats, which may be related to its regulation on brain-gut peptide secretion and improvement of ICC function. The EES significantly increased the serum ghrelin and GAS concentration, reduced the production of SS and VIP, increased the number of ICC through the c-Kit/SCF signaling pathway, so as to improve the slow-wave production of the stomach and regulate the rhythm of smooth muscle contraction activity. However, more in-depth experimental studies may improve understanding of other molecular pathways in the treatment of DGP with EES, and prospective clinical studies are expected to assess its efficacy and safety.
